# Assessment of Resident and AI Chatbot Performance on the University of Toronto Family Medicine Residency Progress Test: Comparative Study

**DOI:** 10.2196/50514

**Published:** 2023-09-19

**Authors:** Ryan ST Huang, Kevin Jia Qi Lu, Christopher Meaney, Joel Kemppainen, Angela Punnett, Fok-Han Leung

**Affiliations:** 1 Temerty Faculty of Medicine University of Toronto Toronto, ON Canada; 2 Department of Family and Community Medicine University of Toronto Toronto, ON Canada; 3 Division of Haematology The Hospital for Sick Children Toronto, ON Canada

**Keywords:** medical education, medical knowledge exam, artificial intelligence, AI, natural language processing, NLP, large language model, LLM, machine learning, ChatGPT, GPT-3.5, GPT-4, education, language model, education examination, testing, utility, family medicine, medical residents, test, community

## Abstract

**Background:**

Large language model (LLM)–based chatbots are evolving at an unprecedented pace with the release of ChatGPT, specifically GPT-3.5, and its successor, GPT-4. Their capabilities in general-purpose tasks and language generation have advanced to the point of performing excellently on various educational examination benchmarks, including medical knowledge tests. Comparing the performance of these 2 LLM models to that of Family Medicine residents on a multiple-choice medical knowledge test can provide insights into their potential as medical education tools.

**Objective:**

This study aimed to quantitatively and qualitatively compare the performance of GPT-3.5, GPT-4, and Family Medicine residents in a multiple-choice medical knowledge test appropriate for the level of a Family Medicine resident.

**Methods:**

An official University of Toronto Department of Family and Community Medicine Progress Test consisting of multiple-choice questions was inputted into GPT-3.5 and GPT-4. The artificial intelligence chatbot’s responses were manually reviewed to determine the selected answer, response length, response time, provision of a rationale for the outputted response, and the root cause of all incorrect responses (classified into arithmetic, logical, and information errors). The performance of the artificial intelligence chatbots were compared against a cohort of Family Medicine residents who concurrently attempted the test.

**Results:**

GPT-4 performed significantly better compared to GPT-3.5 (difference 25.0%, 95% CI 16.3%-32.8%; McNemar test: *P*<.001); it correctly answered 89/108 (82.4%) questions, while GPT-3.5 answered 62/108 (57.4%) questions correctly. Further, GPT-4 scored higher across all 11 categories of Family Medicine knowledge. In 86.1% (n=93) of the responses, GPT-4 provided a rationale for why other multiple-choice options were not chosen compared to the 16.7% (n=18) achieved by GPT-3.5. Qualitatively, for both GPT-3.5 and GPT-4 responses, logical errors were the most common, while arithmetic errors were the least common. The average performance of Family Medicine residents was 56.9% (95% CI 56.2%-57.6%). The performance of GPT-3.5 was similar to that of the average Family Medicine resident (*P*=.16), while the performance of GPT-4 exceeded that of the top-performing Family Medicine resident (*P*<.001).

**Conclusions:**

GPT-4 significantly outperforms both GPT-3.5 and Family Medicine residents on a multiple-choice medical knowledge test designed for Family Medicine residents. GPT-4 provides a logical rationale for its response choice, ruling out other answer choices efficiently and with concise justification. Its high degree of accuracy and advanced reasoning capabilities facilitate its potential applications in medical education, including the creation of exam questions and scenarios as well as serving as a resource for medical knowledge or information on community services.

## Introduction

Technological advances continue to have disruptive impacts on society. One recent example involves the development of artificial intelligence (AI)–based chatbots, deriving from advances in deep learning, natural language processing, transformers, and related large language models (LLMs). These chatbots have been designed to mimic interactive conversations, whereby a user inputs a (potentially complex) query and the chatbot generates a human-like response. Since their inception, these chatbots have been used for a variety of applications, including answering questions, generating explanations and summarizations, translating between languages, and various other tasks involving natural languages [1]. These applications have translated into the integration of LLMs into industries, including consulting, information technology, and education [2]. The first model(s) to gain widespread recognition and adoption were OpenAI’s ChatGPT models, GPT-3.5 and GPT-4, and more recently, a variety of other LLM-based chatbots have been developed, including Google Bard, Facebook Llama, and Anthropic AI’s Claude.

Researchers have recently started to evaluate the performance of these LLM-based chatbots across various domains and have begun to question whether these models demonstrate the qualities of artificial general intelligence [3]. Preliminary evaluations suggest the models have a strong understanding of the semantics and syntax of many natural languages [4] and perform natural language processing tasks [5]. Models have also demonstrated the ability to excel in providing responses to queries related to mathematics, sciences, computer programming, logical reasoning, and humanities [6,7]. Subject matter experts have begun to formally investigate the performance of these LLM-based chatbots on several domain-specific, high-stakes educational examinations in medicine, law, engineering, business or finance, and other areas of the arts and sciences. On the United States Medical Licensing Exam (USLME), GPT-3.5 performed at or near the passing threshold on all 3 exams: Step 1, Step 2 CK, and Step 3 [8]. In many cases, preliminary evidence suggests that these LLMs can oftentimes outperform human subject matter experts across a wide range of high-stakes examinations [9].

The objective of our study is to compare the performance of GPT-3.5 and GPT-4 against medical residents on the formative multiple-choice Progress Test [10] administered to residents training in the University of Toronto’s Family Medicine residency program. The Progress Test consists of case-based knowledge questions emphasizing the assessment of key competency learning points. There has been an ensuing debate about how LLMs may impact the field of education [11]. Comparing GPT-3.5 and GPT-4 performance against medical residents will aim to provide insights into their utility in supporting medical learners.

## Methods

### Study Design and Settings

The University of Toronto Department of Family and Community Medicine Progress Test is a formative assessment, intending to give residents an indication of their progress in the Family Medicine Expert role and help them prepare for Board Certification assessments. It is taken by residents biannually and is formatted as a closed multiple-choice exam developed by content area experts, with each question consisting of 4 response options (labelled as A-D). An official University of Toronto Department of Family and Community Medicine Progress Test was used for this study, consisting of 110 questions. This version of the test was administered to 321 University of Toronto Family Medicine postgraduate year 1 (PGY-1) and 2 (PGY-2) residents on October 19, 2022. Residents were given 4 hours to complete the exam. Out of a total of 110 questions, 2 questions that required the input of images were excluded. A total of 108 questions were included in the study, with an average question length of 1081.56 (SD 282.84) characters (Table 1). The 108 questions were stratified into 11 areas of Family Medicine knowledge, including (1) childhood and adolescent care, (2) elderly care, (3) emergency medicine, (4) end-of-life care, (5) family medicine, (6) in-hospital care, (7) maternity care, (8) mental health, (9) musculoskeletal medicine, (10) surgical skills, and (11) women’s health. Medical resident performance on the test was assessed (N=321) and quantitatively and qualitatively compared against GPT-3.5 and GPT-4.

**Table 1:** Characteristics of the University of Toronto Department of Family and Community Medicine (DFCM) Progress Test.

Exam category	Available questions	Text-based questions	Question length (characters)
Childhood and adolescent care	10	10	1003.5
Elderly care	10	10	1150.5
Emergency medicine	10	10	1065.8
End-of-life care	10	10	1086.1
Family medicine	10	9	937.7
In-hospital care	10	10	1292.2
Maternity care	10	10	958.1
Mental health	10	10	1055.8
Musculoskeletal medicine	10	9	1137.1
Surgical skills	10	10	1087.7
Women’s health	10	10	1136.1
Total	110	108	1081.6

### Question Input and Response Output

Each question was inputted into both GPT-3.5 and GPT-4 exactly as they appeared on the official multiple-choice examination, with multiple-choice response options labelled A-D, across 3 trials. Before the input of each question, ChatGPT was refreshed to clear all previous conversation history, ensuring that the AI chatbot’s responses were not influenced by active conversations. A new ChatGPT Plus account with no previous conversation history was used to ensure that there was no conversation data influencing the study. All questions were inputted exactly as they appeared on the official Progress Test on April 2nd, 2023.

### Response Output and Evaluation Metrics

Each response was independently reviewed by 2 authors (RSH and KJQL) to determine which multiple-choice question was selected, and conflicts were resolved through a third impartial author (FHL). The following data were collected: the date of question input into ChatGPT, the response length in characters, the response length in seconds, whether a rationale was provided for why other responses were not chosen, and the root cause for all incorrect responses. If the AI chatbot chose all of the above or none of the above, then the response was marked as incorrect, as none of the questions had these options as one of the 4 choices. For each question, it was recorded whether the answer explicitly listed reasons why the other options were incorrect, and therefore, not chosen. The root causes of error in incorrect responses were classified into 3 mutually exclusive categories: logical errors, information errors, and arithmetic errors. Logical errors occurred when the AI chatbot attained the relevant information for the question but did not use the information correctly to find the answer. Information errors were classified when the AI chatbot either gathered incorrect information from the question itself or from external sources, leading to an incorrect answer. Arithmetic errors were attributed to mathematical mistakes in calculations. If more than 1 type of error was identified, the response was carefully reviewed to determine which specific cause directly led to the incorrect response made by the AI chatbot.

### Statistical Analysis

We estimated the percentage of correct responses to the Family Medicine Progress Test for GPT-3.5 and GPT-4, respectively. We estimated the percentage of correct responses (and 95% CIs) for the 321 Family Medicine residents using a binomial generalized estimating equation model, with a compound symmetric working correlation structure. We compared whether the point estimates of performance for the LLM-based chatbots (GPT-3.5 and GPT-4, respectively) were contained within the 95% CIs characterizing the average resident performance on the progress test. We used Wald tests (with robust SEs) to compare the LLM-based chatbot performances (GPT-3.5 and GPT-4) against that of the average Family Medicine resident. Similar stratified analyses were conducted for each of the 11 priority areas comprising the test.

We used the McNemar test and Agresti and Min’s [12] confidence interval method to compare the performance of GPT-3.5 versus GPT-4 on the progress test. Paired 2-tailed *t* tests were used to compare GPT-3.5 versus GPT-4 with respect to the mean length and mean time of generated responses. The McNemar test was used to compare GPT-3.5 and GPT-4 on whether a rationale was given for answers provided on response outputs.

All statistical analyses were conducted using R (version 4.3; R Core Team).

### Ethics Approval

Approval for this study was obtained from the University of Toronto research ethics board (Protocol #00044429).

## Results

### Overall Performance

A total of 10 questions were included from each question category, with the exception of family medicine and musculoskeletal medicine, which had 9 questions each (Table 1).

The percentage of correctly answered questions on the Family Medicine Progress Test was 57.4% (62/108) for GPT-3.5, compared to 82.4% (89/108) for GPT-4 (Table 2). The 25.0% (95% CI 16.3%-32.8%) improvement in the percentage of correctly identified answers for GPT-4, compared to GPT-3.5, was statistically significant (McNemar test: *P*<.001).

**Figure 1:**
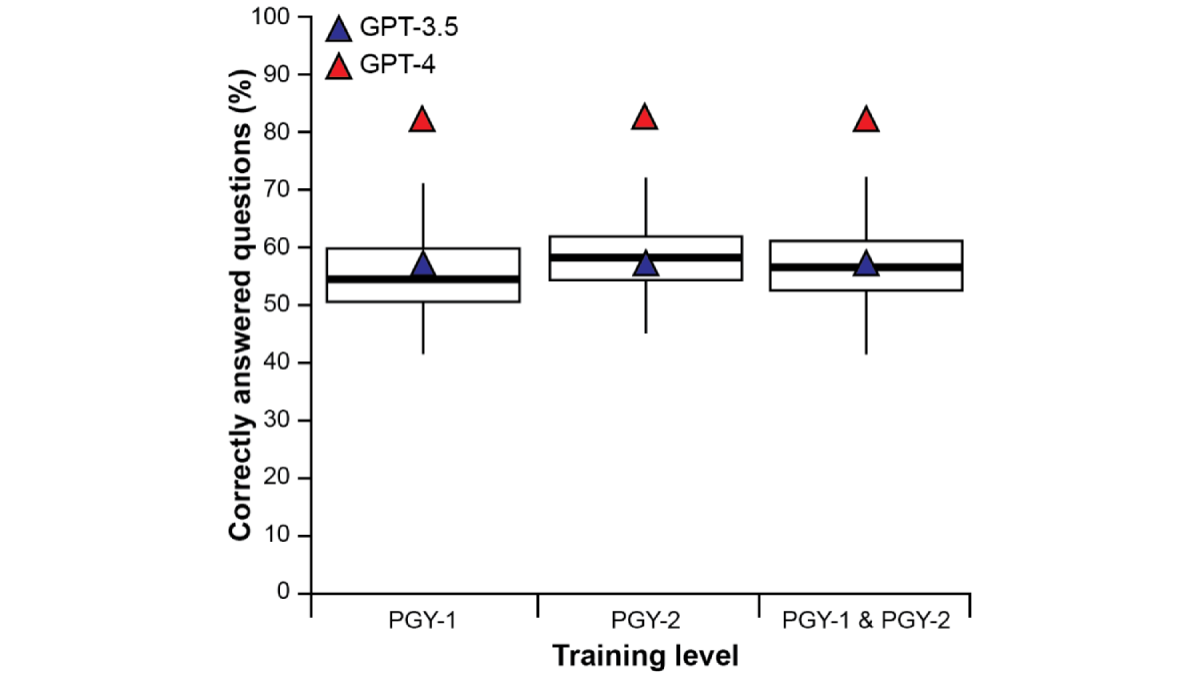
Side-by-side box plots illustrating the percentage of correctly answered Progress Test questions for Family Medicine residents in the postgraduate year 1 (PGY-1) cohort (left), the postgraduate year 2 (PGY-2) cohort (middle), and the combined PGY-1 + PGY-2 cohorts (right). The blue triangles indicate the percentage of correctly answered questions by GPT-3.5. The red triangles indicate the percentage of correctly answered questions by GPT-4.

A total of 321 Family Medicine residents completed the progress test in October 2022. The average performance of Family Medicine residents was 56.9% (95% CI 56.2%-57.6%). The highest-performing resident scored 72.2% (78/108) on the exam. The lowest-performing score was 41.7% (45/108; Figure 1). GPT-3.5 demonstrated performance comparable to that of an average resident in the Family Medicine training program (*P*=.16), whereas the performance of GPT-4 exceeded that of the average resident (*P*<.001) and, in fact, was the top score among all participants who took the examination. Similar inferences were made when the results were stratified according to the level or year of training.

When considering performance stratified according to Family Medicine priority areas, GPT-3.5 and GPT-4 both answered 80% of questions correctly in the childhood and adolescent care category, but GPT-3.5 demonstrated lower performance in every other category, with the lowest performance being in elderly care, with only 30% of the questions answered correctly. GPT-4 performed the best in emergency medicine, mental health, surgical skills, and women’s health, where it answered 90% of the questions correctly, and the lowest performance in end-of-life care and maternity care, with a score of 70%.

**Table 2:** The overall and stratified performance of GPT-3.5, GPT-4, as well as postgraduate year 1 (PGY-1) and postgraduate year 2 (PGY-2) residents on the University of Toronto Department of Family and Community Medicine (DFCM) Progress Test.

Exam Category	Correct answers				
	GPT-3.5, n (%)	GPT-4, n (%)	PGY-1 residents (n=162), percentage (95% CI)	PGY-2 residents (n=159), percentage (95% CI)	PGY-1 and PGY-2 residents (N=321), percentage (95% CI)
Childhood and adolescent care	8 (80.0)	8 (80.0)	62.3 (60.1-64.5)	64.4 (62.3-66.5)	62.3 (60.1-64.5)
Elderly care	3 (30.0)	8 (80.0)	50.0 (47.8-52.2)	50.5 (48.3-52.7)	50.0 (47.8-52.2)
Emergency medicine	7 (70.0)	9 (90.0)	54.0 (51.7-56.3)	57.3 (55.2-59.4)	54.0 (51.7-56.3)
End-of-life care	6 (60.0)	7 (70.0)	50.4 (48.4-52.4)	52.6 (50.5-54.6)	50.4 (48.4-52.4)
Family medicine	5 (55.6)	7 (77.8)	41.0 (38.8-43.3)	47.2 (44.5-49.8)	41.0 (38.8-43.3)
In-hospital care	5 (50.0)	8 (80.0)	63.6 (61.5-65.6)	63.7 (61.5-65.9)	63.6 (61.5-65.6)
Maternity care	4 (40.0)	7 (70.0)	60.7 (58.2-63.2)	68.1 (65.7-70.3)	60.7 (58.2-63.2)
Mental health	6 (60.0)	9 (90.0)	49.3 (47.0-51.6)	51.8 (49.4-54.1)	49.3 (47.0-51.6)
Musculoskeletal medicine	5 (55.6)	8 (88.9)	49.4 (46.9-51.9)	54.8 (52.8-56.8)	49.4 (46.9-51.9)
Surgical skills	7 (70.0)	9 (90.0)	69.4 (67.1-71.5)	71.3 (69.2-73.3)	69.4 (67.1-71.5)
Women’s health	6 (60.0)	9 (90.0)	56.7 (54.5-58.8)	60.6 (58.4-62.7)	56.7 (54.5-58.8)
Total	62 (57.4)	89 (82.4)	55.3 (54.4-56.3)	58.5 (57.6-59.5)	56.9 (56.2-57.6)

### Quantitative and Qualitative Comparison of Responses

GPT-4 took longer to respond to exam questions compared to GPT-3.5 (paired *t* test: *P*<.001; Table 3). The responses generated by GPT-4 were more concise compared to GPT-3.5 (paired *t* test: *P*<.001). GPT-4 was more likely to provide a rationale for the multiple-choice response option selected (McNemar test: *P*<.001; Textbox 1). For both GPT-4 and GPT-3.5, logical and informational errors were the most common, while arithmetic errors were the least frequently observed.

**Table 3:** A comparison of GPT-3.5 and GPT-4 with respect to response generation time, response length, rationale for selected responses, and the types of errors committed.

Characteristics			GPT-3.5		GPT-4
Response time (sec), mean (95% CI)			10.5 (9.8-11.3)		28.0 (26.0-30.0)
Response length (characters), mean (95% CI)			1011 (929-1092)		787 (722-852)
Rationale for other answer options, n (%)			18 (16.7)		93 (86.1)
**Reason for error^a^, n (%)**					
	Logical error	22 (47.8)		11 (57.9)	
	Arithmetic error	5 (10.9)		1 (5.3)	
	Information error	19 (41.3)		7 (36.8)	

^a^Reasons for error were calculated only for incorrect responses.

**A sample surgical skills multiple-choice question with associated GPT-3.5 and GPT-4 outputs.**

**Source question:**

You are a family physician working in a remote rural ER [emergency room]. A 52-year-old male presents with a facial injury. He accidentally cut his right cheek with a hunting knife several hours prior when he was out hunting. His past medical history is unremarkable. He is not on any medications. His tetanus status is up to date. On examination, the patient has normal vitals. You observe a 2 cm laceration on his right cheek that penetrates through to the oral mucosa. The wound edges are clean and clear. There is minimal surrounding erythema. The remainder of your physical exam is unremarkable. You repair the lesion. How should you manage this patient's after-care?

A. Oral antibiotics (correct answer)

B. Alcohol mouthwash rinses

C. Follow-up with dentistry in 7 days

D. Daily external wound cleansing with hydrogen peroxide

**GPT-3.5’s response:**

B. Alcohol mouthwash rinses.

In this case, the patient has a laceration on his cheek that penetrates through to the oral mucosa. While the wound edges are clean and clear, there is a risk of infection due to the location of the wound and the potential for contamination from the hunting knife.

After repair of the wound, it is appropriate to provide after-care instructions to the patient. Alcohol mouthwash rinses are recommended to the patient as they have antiseptic properties and can help reduce the risk of infection. Antibiotics are not indicated in this case, as the wound appears clean and there is no evidence of infection at this time. The patient should be instructed to rinse his mouth with the mouthwash several times a day for the next several days.

**GPT-4’s response:**

A. Oral antibiotics.

For this patient with a facial laceration that penetrates through to the oral mucosa, the appropriate after-care management would be oral antibiotics. Since the laceration communicates with the oral cavity, there is an increased risk of infection due to the bacteria present in the mouth. Prescribing oral antibiotics can help reduce the risk of infection. Additionally, the patient should be advised to maintain good oral hygiene and to avoid any activities that may strain or disrupt the wound while it is healing.

Using alcohol mouthwash rinses (B) could cause pain and irritation in the wound area. Hydrogen peroxide (D) for external wound cleansing is not recommended, as it may delay wound healing and cause tissue damage. Follow-up with dentistry in 7 days (C) may not be necessary unless there are concerns about dental injury or ongoing oral issues related to the wound.

## Discussion

### Principal Findings

This was the first study to evaluate the performance of GPT-3.5 and GPT-4 on a Family Medicine resident assessment test (ie, in the context of a Canadian medical education setting). The average Family Medicine resident completing the progress test in October 2022 scored 56.7%, comparable to that of GPT-3.5. GPT-4 performed significantly better than GPT-3.5 and the average Family Medicine resident (PGY-1 or PGY-2) on the Progress Test. Remarkably, the performance of GPT-4 exceeded the best-performing medical resident score from the October 2022 test sitting. For reference, residents are provided 4 hours to complete the exam. The cumulative time it takes for GPT-4 to complete the exam, calculated as the product of its mean response time and the number of questions, is about 18 minutes.

Several previous studies have examined the performance of GPT-3.5 and GPT-4 in the context of high-stakes medical knowledge and licensing examinations. Similar to the findings in our study that highlight GPT-4’s superior performance over GPT-3.5, GPT-4 demonstrated considerable improvement compared to GPT-3.5 in its performance on a sample United States Medical Licensing Examination (USMLE) Step 3 exam [13], Japanese Medical Licensing Examination [14], and Korean National Licensing Examination [15] for traditional Korean medicine.

GPT-3.5’s performance was relatively poor in the exam categories of elderly care and maternity care. Geriatric patient cases are often characterized by patients with greater medical complexity, and their disease may manifest with subtler or atypical symptoms [16]. Maternity care is a highly diverse field that spans from prenatal care to postpartum care, with each stage embodying unique clinical nuances. GPT-4’s relatively better performance in these respective exam categories and across most exam categories is likely attributable to its broader knowledge base and stronger clinical reasoning skills. GPT-4's refined performance is believed to stem from its increased size and architecture, as it has been trained on a larger data set and is estimated to have significantly more model parameters.

To examine why GPT-4 excels over GPT-3.5, we provide an example of GPT-3.5’s incorrect response against GPT-4’s correct response to a sample test question (Textbox 1). This question was selected because it tests the critical concept of infection risk management, emphasizes GPT-4’s broader knowledge base and stronger reasoning ability, and highlights a well-known weakness of LLMs. In the sample test question, both GPT-3.5 and GPT-4 identify a risk of infection for a patient who presents with a cheek laceration that penetrates the oral mucosa with minimal signs of infection. However, GPT-3.5 makes a logical error by suggesting the use of an alcohol mouthwash, as the wound does not appear actively infected. The model generated an incorrect response with justification based on information that cannot be verified by the source content, which represents a phenomenon that is termed an (extrinsic) “hallucination” [17]. Unsurprisingly, hallucinations raise concerns about a model’s integrity and overall accuracy [18] and may mislead learners into believing an incorrect response to be correct. Accordingly, evaluation of model performances on specific academic tests or tasks is necessary to provide insights into their strengths and weaknesses. Compared to GPT3.5, GPT-4’s response explains in greater detail that there is an increased infection risk because of the oral cavity wound communication. GPT-4 not only chooses and justifies its correct response, antibiotics, but it also describes additional management guidelines that were not prompted in the initial multiple-choice question. GPT-4’s response even further justifies why it did not select the other incorrect choices. Its response can be improved by citing available literature that supports evidence-based practice.

Although previous studies have highlighted GPT-4’s improved performance compared to GPT-3.5 on medical assessments, we believe that GPT-4’s superior test performance against medical residents on the Progress Test substantiates its credibility toward becoming a valuable medical education tool for medical residents at different levels of performance and training. Different roles for LLM-based chatbots in medical education include content creation, such as test questions or case-based scenarios [19]. Test creators, including faculty and preceptors, spend considerable time and resources to produce satisfactory questions [20]. Family Medicine residency training programs often use simulated structured clinical examinations as both low- and high-stakes assessment tools [21]. Through future rigorous studies that include addressing LLM weaknesses, such as hallucinations, LLMs may eventually serve as a cost-effective method to generate case scenarios appropriate for the training level of a Family Medicine resident. LLM-based chatbots can also assist in both individual and small-group learning. Our study showed that GPT-4 provided a rationale for most of its response choices on the Progress Test. As our Family Medicine Faculty experts have created and possess an official answer key to the Progress Test, LLM responses, either correct or incorrect, can be referenced against the answer key. This provides insights into the type of questions for which LLMs may provide similar clinical reasoning to that of a clinical expert and, in contrast, when and how LLMs may commit errors in their clinical reasoning. Error frequency, type, and severity, depending on the LLM's sophistication, can be used by preceptors to identify possible clinical reasoning pitfalls that medical learners may encounter. Trainees can also leverage this information to supplement their learning by comparing the structure of their clinical reasoning processes against the rationale of the AI. Given the comprehensive nature and broad scope of Family Medicine, it would be beneficial for trainees to have an accessible tool with a vast knowledge base, allowing them to quickly ask questions about a variety of medical concepts. Similar roles for LLMs exist in group learning [22], either through case-based learning or didactic teaching sessions, which are often scheduled at regular intervals throughout a Family Medicine residency program curriculum. Family Medicine practitioners and trainees also serve as a bridge between patients and community resources [23]. LLMs efficiently summarize lists of community programs or organizations that residents can learn more about to help them decide how to best coordinate patient care.

### Limitations

Our study presents several limitations. Development of LLMs is progressing rapidly, and our study only includes the comparison of OpenAI’s GPT-3.5 against GPT-4. A comparison that encompasses other LLM models, including but not limited to Google Bard, Facebook Llama, and Anthropic’s Claude, would ultimately provide stronger insights into determining which LLM is best suited for the medical education training program. We also did not have access to GPT-3.5 or GPT-4 application programming interfaces. Additionally, GPT-3.5 and GPT-4 are subject to continuous updates supplemented by user feedback and server latency. We tried to restrict these effects by inputting all multiple-choice questions into the LLMs on the same day and double-checking that the chatbot gave the same multiple-choice answers to each question in 2 different web browsers. Our results should be interpreted in the timeframe that it was achieved, as GPT-3.5 and GPT-4 performance will likely continue to improve over time.

The Progress Test used in this study represents only 1 iteration of the Progress Test examination, with participation from only one cohort of Family Medicine residents. A larger sampling of Progress Test questions and resident performance may have been obtained if multiple iterations of the Progress Test on different examination sitting dates were used. Questions on the formative Progress Test are also all multiple-choice based.

Future work should evaluate the performance of LLMs on different types of assessment questions, including rank-based and open-ended questions. As our study and several other studies evaluate both the quantitative and qualitative performance of models on medical knowledge examinations, it would be beneficial to appraise the suitability of various LLM evaluation frameworks. Ultimately, future studies should assess the short- and long-term effectiveness of integrating LLM applications into medical education.

### Conclusions

As AI sophistication continues to grow, our study shows that GPT-4 significantly outperforms GPT-3.5 as well as PGY-1 and PGY-2 medical residents, including the top-scoring resident, on a medical knowledge multiple-choice Progress Test designed for Family Medicine residents. GPT-4 demonstrates a broad knowledge base and strong reasoning abilities in its responses, as evidenced by its high level of accuracy and logical justification for response choices. Accordingly, there is great potential to integrate GPT-4 as an innovative learning tool in a Family Medicine residency program. Some applications include creating questions and scenarios for medical learner assessments, supplementing medical knowledge, and generating informational lists of community resources to help residents in coordinating care.

## Abbreviations

AI: artificial intelligence
LLM: large language model
PGY-1: postgraduate year 1
PGY-2: postgraduate year 2
USMLE: United States Medical Licensing Exam
